# Brain Health PRO: Evaluating a Web‐Based Multidomain Intervention to Improve Dementia Literacy and Self‐Efficacy

**DOI:** 10.1002/alz70860_103191

**Published:** 2025-12-23

**Authors:** Sylvie Belleville, Nicole D. Anderson, John R. Best, Paul W.H. Brewster, January Durant, Mohamed Abdelhafid Kadri, Andrew Lim, Jody‐Lynn Lupo, Manuel Montero‐Odasso, Haakon B. Nygaard, Penelope J. Slack, Howard Chertkow, Howard H. Feldman, CCNA‐CAN‐THUMBS UP Study Group

**Affiliations:** ^1^ Institut Universitaire de Gériatrie de Montréal, Montréal, QC, Canada; ^2^ Rotman Research Institute, Toronto, ON, Canada; ^3^ Simon Fraser University, Vancouver, BC, Canada; ^4^ University of British Columbia, Vancouver, BC, Canada; ^5^ Cognition & Technology Research Group, Institute on Aging and Lifelong Health, University of Victoria, Victoria, BC, Canada; ^6^ University of Victoria, Victoria, BC, Canada; ^7^ Department of Neurosciences, University of California San Diego, La Jolla, CA, USA; ^8^ University of California San Diego, La Jolla, CA, USA; ^9^ Alzheimer's Disease Cooperative Study, University of California San Diego, La Jolla, CA, USA; ^10^ Centre de recherche de l’Institut Universitaire de gériatrie du CIUSSS du Centre‐Sud‐de‐l’Île‐de‐Montréal, Montréal, QC, Canada; ^11^ Sunnybrook Research Institute, Toronto, ON, Canada; ^12^ Gait and Brain Laboratory, Canada, ON, Canada; ^13^ Schulich School of Medicine & Dentistry, Division of Geriatric Medicine, Western University, London, ON, Canada; ^14^ Rotman Research Institute, Baycrest Health Sciences, Toronto, ON, Canada; ^15^ University of California San Diego, Department of Neurosciences, La Jolla, CA, USA

## Abstract

**Background:**

Early interventions addressing lifestyle‐related risk factors present a compelling strategy for mitigating cognitive decline. In response to this need, the Canadian Consortium on Neurodegeneration in Aging has developed Brain Health Pro, an innovative remote web‐based educational intervention aimed at enhancing dementia literacy and supporting older adults in adopting healthier lifestyles. The program focuses on seven key topics that influence brain health: physical activity, diet, mental stimulation, sleep, social and psychological health, vascular health, and vision and hearing.

**Method:**

This is a twelve‐month prospective multi‐center longitudinal study testing the effect of Brain Health PRO. The intervention comprises 181 chapters delivered progressively over 10 months. Each chapter is interactive, focusing on a specific topic, and provides relevant information, practical tips, and guidelines. The content is presented based on priorities tailored to individual risk levels and personal preferences. Participants included older adults with no cognitive impairment or those with mild cognitive impairment and at least one dementia risk factor (first‐degree family history of dementia, hypertension, hypercholesterolemia, body mass index >30 or physical inactivity). The primary outcome was dementia literacy, assessed using the Alzheimer's Disease Knowledge Scale at 6 and 12 months. Secondary outcomes included self‐efficacy, measured by the General Self‐Efficacy Scale, and attitudes toward dementia, assessed using the PRISM‐PC questionnaire.

**Result:**

A covariate‐adjusted linear mixed model for the intention‐to‐treat sample (*N* = 353; mean age = 69.7) revealed a significant positive change in dementia literacy at 6 months (standardized change = 0.246; *p* = 0.012) and a significant improvement in self‐efficacy at 12 months (standardized change = 0.157; *p* = 0.008). However, no significant change was observed in attitudes toward dementia.

**Conclusion:**

Brain Health Pro offers a personalized and accessible approach that can increase individuals' knowledge about dementia risk while enhancing their general self‐efficacy. These dimensions are critical determinants of health. Improving them may foster long‐lasting lifestyle changes to support sustained cognitive health.

